# New Alternative Mixtures of Cryoprotectants for Equine Immature Oocyte Vitrification

**DOI:** 10.3390/ani11113077

**Published:** 2021-10-28

**Authors:** Daniel Angel-Velez, Tine De Coster, Nima Azari-Dolatabad, Andrea Fernandez-Montoro, Camilla Benedetti, Osvaldo Bogado Pascottini, Henri Woelders, Ann Van Soom, Katrien Smits

**Affiliations:** 1 Department of Reproduction, Obstetrics and Herd Health, Faculty of Veterinary Medicine, Ghent University, Salisburylaan 133, 9820 Merelbeke, Belgium; tine.decoster@ugent.be (T.D.C.); nima.azaridolatabad@ugent.be (N.A.-D.); andrea.fernandezmontoro@ugent.be (A.F.-M.); camilla.benedetti@ugent.be (C.B.); osvaldo.bogado@ugent.be (O.B.P.); ann.vansoom@ugent.be (A.V.S.); katrien.smits@ugent.be (K.S.); 2 Research Group in Animal Sciences—INCA-CES, Universidad CES, Medellin 050021, Colombia; 3 Veterinary Physiology and Biochemistry, Department of Veterinary Sciences, University of Antwerp, 2610 Wilrijk, Belgium; 4 Wageningen Livestock Research, Wageningen University and Research, Droevendaalsesteeg 1, 6708 PB Wageningen, The Netherlands; Henri.Woelders@wur.nl

**Keywords:** cryoprotective agents, equine, ICSI, oocyte, vitrification, warming

## Abstract

**Simple Summary:**

Oocyte cryopreservation allows female gametes to be conserved for long periods, which would be of benefit for mares of high genetic merit, but its efficiency is not satisfactory yet. Therefore, the aim of this study was to optimize a vitrification protocol for equine oocytes using a systematic approach. We performed a side-by-side comparison of different cryoprotective agents (CPAs) during the vitrification and warming of equine oocytes. In the first experiment, a fixed mixture of CPAs that enter the oocyte was used, and three sugars were compared, which cannot penetrate the oocyte but provide protection through an osmotic effect. In the second experiment, one sugar from the first experiment was selected to compare three mixtures of CPAs that enter the oocyte. Overall, the embryo development was reduced after oocyte cryopreservation when compared to fresh oocytes. Yet, we were able to produce embryos with all six cryoprotective agent mixtures, and we identified one promising combination of cryoprotectants, consisting of propylene glycol, ethylene glycol, and galactose, that resulted in blastocyst rates in the same range as the fresh control group.

**Abstract:**

Equine oocyte vitrification would benefit the growing in vitro embryo production programs, but further optimization of the protocol is necessary to reach clinical efficiency. Therefore, we aimed to perform a direct comparison of non-permeating and permeating cryoprotective agents (CPAs) during the vitrification and warming of equine immature oocytes. In the first experiment, cumulus oocytes complexes (COCs) were vitrified comparing sucrose, trehalose, and galactose in combination with ethylene glycol (EG) and dimethyl sulfoxide (DMSO). In the second experiment, the COCs were vitrified using three mixtures of permeating CPAs in a 50:50 volume ratio (ethylene glycol-dimethyl sulfoxide (ED), propylene glycol-ethylene glycol (PE), and propylene glycol-dimethyl sulfoxide (PD)) with galactose and warmed in different galactose concentrations (0.3 or 0.5 mol/L). Overall, all the treatments supported blastocyst formation, but the developmental rates were lower for all the vitrified groups in the first (4.3 to 7.6%) and the second (3.5 to 9.4%) experiment compared to the control (26.5 and 34.2%, respectively; *p* < 0.01). In the first experiment, the maturation was not affected by vitrification. The sucrose exhibited lower cleavage than the control (*p* = 0.02). Although the galactose tended to have lower maturation than trehalose (*p* = 0.060) and control (*p* = 0.069), the highest numerical cleavage and blastocyst rates were obtained with this CPA. In the second experiment, the maturation, cleavage, and blastocyst rates were similar between the treatments. Compared to the control, only the ED reached similar maturation (*p* = 0.02) and PE similar cleavage (*p* = 0.1). The galactose concentration during warming did not affect the maturation, cleavage, or blastocyst rates (*p* > 0.1), but the PE-0.3 exhibited the highest blastocyst rate (15.1%) among the treatments, being the only one comparable to the control (34.2%). As such, PE–galactose provides a valuable option for equine immature oocyte vitrification and should be considered for the future optimization of the protocol.

## 1. Introduction

Oocyte cryopreservation allows conserving both female genetics and fertility. It has gained increased interest during the last few years in the horse, especially as a complement for assisted reproductive techniques (ARTs), like ovum pick-up (OPU), intracytoplasmic sperm injection (ICSI), and cloning. The application of these ARTs has increased dramatically due to the rise in their efficiency and the consistent pregnancy rates that can be achieved [1,2,3,4]. Gamete cryopreservation would further increase ICSI flexibility by allowing to postpone the decision on the fertilizing stallion. Moreover, oocytes could be salvaged from recently deceased animals in places where ICSI is not available; oocytes might be collected and stored outside the reproductive season, and good-quality germ lines of young mares could be preserved before they are enrolled in competition [5,6]. Finally, horse oocyte banks would allow the worldwide spread of valuable female genetics for commercial purposes, for breeding programs of endangered equid breeds and species, or for research in any ART in horses [5,7]. However, the mammalian oocyte is one of the hardest cells to cryopreserve [8], and, up to now, the efficiency of equine oocyte cryopreservation is limited. Only a few foals have been born resulting from mature or immature oocytes cryopreserved by vitrification [9,10]. The vitrification of immature and in vitro matured equine oocytes compromises embryo development after ICSI [11,12,13,14]. Hence, in order to exploit the application potential of equine oocyte vitrification, further optimization of the vitrification protocols is necessary (for review see De Coster & Angel-Velez, et al., 2020 [5]).

During vitrification, high concentrations of cryoprotective agents (CPAs) (30–40% *v*/*v*) combined with a high cooling rate are required to prevent intra- and extracellular ice formation and achieve stable vitrification [15,16]. Both permeating and non-permeating solutes may be used as CPAs. Permeating CPAs, such as ethylene glycol (EG), dimethyl sulfoxide (DMSO), propylene glycol (PG), and glycerol, are small molecules that can penetrate the plasma membrane and form hydrogen bonds with water molecules to lower the freezing point, which, combined with high cooling and warming rates, can prevent significant intracellular and extracellular ice formation during vitrification and warming [17]. While these actions largely depend on the colligative properties of the CPAs, the effective protection provided by the specific permeating CPAs varies depending on their membrane permeability and other properties, as well as on the maturation stage of the oocyte and the temperature at which they are introduced [18]. However, high concentrations of CPAs can also have toxic effects, and an optimal balance between protection against cryoinjury and toxicity needs to be established. Combining two or more permeating CPAs can decrease the overall CPA toxicity because of the lower concentrations of each CPA used [19,20]. This was also demonstrated by Szurek and Eroglu, 2011 [21] in mouse oocytes, in which 1.5 M PG induced a significant increase in oocyte degeneration (54.2%), while it was possible to avoid the toxicity of PG by decreasing its concentration to 0.75 M and combining it with 0.75 M DMSO. Likewise, Somfai et al. [22] showed that the combination of PG and EG provided greater embryo development after the vitrification of the germinal vesicle (GV)-stage porcine oocytes than did either CPA alone. In mature human oocytes, EG has approximately half the permeability of PG and DMSO, but it is preferred due to its low toxicity [18]. In the horse, EG was used in combination with non-permeating CPAs to preserve oocytes [23]. However, it was not until combinations of EG–DMSO–sucrose [10] and PG–EG–trehalose [11,12] were used that blastocyst development was obtained from vitrified immature equine oocytes. Despite this progress, the blastocyst rates from vitrified equine oocytes remain severely affected, and the optimization of the protocol is compulsory. Only one study has directly compared protocols using different CPAs in the horse, in which PG –EG–trehalose seemed to be the most effective combination [11], but toxicity was still present and no direct comparisons between CPAs at the same concentrations were performed.

Non-permeating CPAs are solutes that do not penetrate the ooplasm and remain in the extracellular compartment during cooling to promote glass formation [24]. Non-permeating CPAs include sugars (e.g., sucrose and trehalose), macromolecules (e.g., Ficoll and bovine serum albumin), and synthetic (co)polymers, such as synthetic ice blockers (e.g., polyvinylpyrrolidone, polyvinyl alcohol, and SuperCool X-1000) [25]. Non-permeating CPAs may be less cytotoxic than permeating ones, although they may result in more mechanical stress from the increased shrinking of cells. The addition of non-permeating CPAs to the vitrification media contributes to the viscosity and tonicity, allowing lower concentrations of permeating CPAs to be used without compromising the vitrification properties [26]. In equine oocyte vitrification, sucrose is the commonly used non-permeating CPA [9,10,27,28,29]. However, trehalose has recently shown promising results. Clerico et al., 2021 [14] reached an encouraging blastocyst rate of 15% (9/56) using trehalose combined with EG–DMSO. Previously, Canesin et al., 2017 [11] compared different treatments and obtained the highest vitrification efficiency with a vitrification medium containing trehalose (42% maturation (10/24), 80% cleavage (8/10), and 10% blastocysts (1/10)), whereas no blastocysts were obtained from sucrose-containing media [11], but, in the latter study, the extracellular CPAs compared had different molar concentrations. In a study in which equal molar concentrations of non-permeating CPAs were compared, a similar pronuclear formation was found for horse oocytes vitrified in a DMSO–EG medium containing sucrose or trehalose [30]. In humans and mice, the oocyte vitrification in a trehalose-containing medium was also associated with higher blastocyst rates than vitrification with sucrose [31], but the differences were not always significant [32,33], probably due to variations in the protocols and concentrations. Besides these disaccharides, which have been the only non-permeating CPAs tested in equine oocytes vitrification up to now (for review, De Coster & Angel-Velez, et al., 2020 [5]), monosaccharides may provide a valuable alternative. Monosaccharides (galactose, fructose, glucose) seem to be more effective osmotic buffers than disaccharides during vitrification and warming [34], and they have been used successfully for the vitrification of bovine [35] and feline [36] oocytes, as well as for bovine [37], porcine [38], dromedary camel [39], alpaca [40], and equine embryos [37,41,42].

The warming procedure is as crucial in oocyte survival as vitrification, and relatively little attention has been paid to the warming systems in all species. During the thawing procedure, the oocyte should slowly recover its original volume to avoid osmotic shock or over-swelling [43]. Commonly, hyperosmolar solutions (1 mol/L) with mono- and disaccharides have been used in warming solutions as an osmotic counterforce in restricting water permeation into the oocyte, preventing a swelling injury [44,45,46]. However, oocytes should be removed from this solution before they start shrinking excessively, which may start (e.g., after 60 s) after sufficient CPA has left the cells and the continued efflux of CPA is accompanied by the efflux of water as a result of the extracellular hyperosmotic solution [47,48]. Direct warming to an iso-osmolar base medium (0 mol/L sugar) has been tried [12]; still, this could create an over-swelling due to the fast inward diffusion of water by osmosis [49]. Therefore, different warming concentrations should be explored to further optimize the warming procedure.

A superior standard protocol describing all the aspects of oocyte cryopreservation has not yet been identified in the horse. Multiple factors influence the success of equine oocyte vitrification, and one crucial technical aspect is the type of CPA [5]. Therefore, the aim of the present study was to determine the optimal combination of CPAs for the vitrification of immature equine oocytes. To do so, we compared (1) three sugars as non-permeating CPAs (sucrose vs. trehalose vs. galactose) with EG and DMSO as permeating CPAs, and (2) three combinations of permeating CPAs (EG–DMSO vs. PG–EG vs. PG–DMSO) with galactose as a non-permeating CPA. Moreover, the efficiency of two different concentrations of galactose in the warming solution (0.3 vs. 0.5 mol/L) was assessed.

## 2. Materials and Methods

### 2.1. Media and Reagents

Dulbecco’s Modified Eagle Medium Nutrient Mixture F-12 (DMEM/F-12), Tissue Culture Medium-199 (TCM-199) with Hanks’ salts, and Tissue Culture Medium-199 with Earle’s salts were purchased from Life Technologies, Merelbeke, Belgium. Unless otherwise stated, all other components were obtained from Sigma, Bornem, Belgium.

### 2.2. Collection of Equine Immature Oocytes

Equine ovaries were obtained from a local slaughterhouse and transported in an insulated box to the laboratory at room temperature within 1 h. All follicles between 5 and 30 mm were aspirated using a 16-gauge needle attached to a vacuum pump (100 mm Hg), scraped with the aspirating needle, and flushed with prewarmed flushing medium (Equiplus, Minitube, Tiefenbach, Germany). The aspirated fluid was collected in sterilized glass bottles, and the bottom content was pipetted several times to a 100/20 mm petri dish until no more oocytes were found. All cumulus–oocyte complexes (COCs) were recovered in TCM-199 with Hank’s salts (Gibco), washed twice, and pipetted with a 200 µm denudation tip (EZ-tip, Origio, Vreeland, the Netherlands) to remove the outer cumulus cells, leaving the corona radiata. Then, COCs were randomly assigned to vitrification or in vitro maturation (IVM; control). Denuded, partially denuded, and clearly expanded COCs surrounded by a hyaluronan-rich matrix were excluded from all experiments.

### 2.3. Oocyte Vitrification and Warming

**Figure 1:**
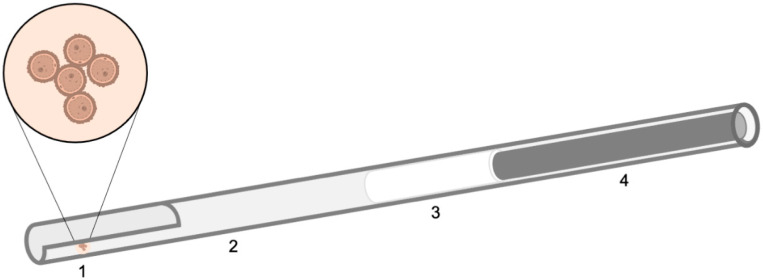
Schematic representation of the custom-made minimal volume (<1 µL) cryo-device. 1: Droplet of vitrification medium with 4 to 6 oocytes. 2: 0.25 mL straw with a cut in one end to allow loading of the oocytes in a minimal volume (<1 μL). E: Cotton of the straw pushed to the middle by the wire. 4: Metal wire, obtained from a paper clip, was inserted to prevent floating in LN2.

The composition of the vitrification and warming solutions is described below in the experimental design and summarized in Table 1. For all experiments, vitrification and warming steps were performed on a heated plate at 39 °C. The vitrification method and the device used for vitrification were based on the “short vitrification protocol” described by Ortiz-Escribano et al., [10] with minor modifications. All oocytes assigned to different vitrification treatments were transferred to a small petri dish (35/10 mm) with 4 mL of base solution (BS), which was equal for all experiments (TCM-199 with Hank’s salts supplemented with 0.4% (*w*/*v*) bovine serum albumin (BSA) (A6003)). Then, four to six oocytes at a time were placed and washed thorough two droplets of 100 µL of equilibration solution (ES) for 25 s. Finally, oocytes were transferred to a 100 µL droplet of vitrification solution (VS) for 15 s, loaded onto a custom-made minimal volume (<1 µL) cryo-device (Figure 1), and plunged into liquid nitrogen. The time between the placement of oocytes in the VS and the immersion of the device into the liquid nitrogen was 30–45 s. Oocytes were loaded using a 200 µm pipette to minimize the volume surrounding the oocytes. Moreover, extra medium surrounding the oocytes was removed with the pipette by capillarity. After at least one week of storage in liquid nitrogen, oocytes were warmed. For warming, the cryo-device was transferred into 4 mL of warming solution 1 (W1), containing BS supplemented with 0.3 or 0.5 mol/L sugar, depending on the experiment, for 5 min. Then, all oocytes were moved to BS until the warming of all oocytes was completed (Table 1).

### 2.4. In Vitro Maturation and ICSI

Vitrified–warmed or fresh oocytes were transferred to maturation medium (TCM-199 with Earl’s salts (Gibco) containing 10% (*v*/*v*) FBS (Gibco), 9.4 μg/mL follicle-stimulating hormone, and 1.88 μg/mL luteinizing hormone (Stimufol, Reprobiol, Ouffet, Belgium)). Maturation was performed in groups of 20–40 COCs in 500 μL maturation medium under paraffin oil (Cooper Surgical, Venlo, The Netherlands). Oocytes were matured at 38.5 °C in 5% CO_2_ in air for 30 h on average (min: 27.5 h; max 32.5 h). Frozen-thawed semen from a single stallion of proven fertility was used for ICSI. Spermatozoa were selected using a 45–90% Percoll density gradient centrifugation for 40 min at 750× *g* at 26 °C. After removal of the supernatant, the sperm pellet was washed in 5 mL of G-MOPS (Vitrolife, Londerzeel, Belgium) and centrifugated for 10 min at 400× *g* at 26 °C. The supernatant was removed again, and the sperm pellet was resuspended in 300 μL of G-MOPS and kept at room temperature until used for ICSI. Mature oocytes (MII), indicated by an extruded polar body, were injected by piezo drill, and presumptive zygotes were cultured as described by Papas et al., 2021 [4]. Cleavage rate was evaluated 2 or 3 days after ICSI, and blastocyst development was monitored daily from day 7 until 10 post ICSI.

### 2.5. Experimental Design

#### 2.5.1. Experiment 1: Effect of Sucrose, Trehalose, or Galactose as Non-Permeating CPAs in VS and in WS on Maturation, Cleavage, and Blastocyst Rates

COCs were obtained as described above and immediately vitrified in three groups using 0.5 M of one of three sugars in the VS: sucrose (S1888; *n* = 155), trehalose (T0167; *n* = 160), or galactose (G5388: *n* = 153). For this experiment, ES contained BS with 10% (*v*/*v*) EG (#102466) and 10% DMSO (#D2650), and VS contained BS with 20% EG, 20% DMSO, and 0.5 M of each sugar (sucrose, trehalose, or galactose). During vitrification, the groups were alternated to keep the oocyte handling time similar. All oocytes were warmed with 0.5 mol/L of the sugar (sucrose, trehalose, or galactose) in WM (Table 1). A control group (*n* = 173) with non-vitrified oocytes was included in every replicate (five replicates). Oocytes of each treatment were warmed consecutively and placed immediately in IVM. The order of warming was considered for the ICSI to keep the maturation duration equal for all groups.

#### 2.5.2. Experiment 2: Effect of Three Different Mixtures of Permeating CPAs and Two Different Warming Regimens on Maturation, Cleavage, and Blastocyst Rates

COCs were vitrified as described above using three different permeating CPA mixtures in a 50:50 ratio: EG–DMSO (ED), PG (#P4347)–DMSO (PD), and PG–EG (PE) (Table 1). For vitrification, ES consisted of BS with 20% (*v*/*v*) of the CPA mix, and VS of BS with 40% (*v*/*v*) of the CPA mix and 0.5 M galactose. For warming, two different concentrations of galactose were used, resulting in six groups (ED-0.5 (*n* = 110), ED-0.3 (*n* = 85), PD-0.5 (*n* = 115), PD-0.3 (*n* = 79), PE-0.5 (*n* = 107), and PE-0.3 (*n* = 90)). A control group (*n* = 242) with non-vitrified oocytes was included in every replicate (four replicates).

### 2.6. Statistical Analysis

All statistical analyses were performed using R-core (version 3.6.1; R Core Team, Vienna, Austria). The oocyte/zygote/embryo was considered as the unit of interest. Generalized mixed effects models were used to analyze the data. In the first experiment, we evaluated the effect of the three sugars as non-permeating CPAs (control vs. sucrose vs. trehalose vs. galactose) on maturation, cleavage, and blastocyst development. In the second experiment, the effects of the CPA mixture (control vs. ED vs. PD vs. PE), the galactose concentration in the WM (0.5 mol/L vs. 0.3 mol/L), and their interaction on maturation, cleavage, and blastocyst rates were assessed. Cleavage and blastocyst rates represent the percentage of cleaved embryos or blastocysts, respectively, per injected oocyte. The control group was resampled randomly to balance lower numbers of injected oocytes in cleavage and blastocyst rate of the interaction (ED-0.5 vs. ED-0.3 vs. PD-0.5 vs. PD-0.3 vs. PE-0.5 vs. PE-0.3 vs. control). For all the models, the replicate was set as random. Results are expressed as least square means and standard errors. The differences between treatment groups were assessed using Tukey’s post hoc test. The significance and tendency levels were set at *p* < 0.05 and *p* < 0.1, respectively.

## 3. Results

### 3.1. Experiment 1: Effect of Non-Permeating CPAs on Maturation, Cleavage, and Blastocyst Rates

#### 3.1.1. Comparison among All Vitrified and Non-Vitrified Oocytes

Overall, when we compare all the oocytes vitrified with EG–DMSO and different sugars as one group, they exhibited maturation rates (51.0 ± 2.5%) similar to fresh oocytes (56.9 ± 3.9%; *p* = 0.19). However, the cleavage (75.3 ± 4.7%) and blastocyst rates (26.5 ± 5.7%) were higher in the fresh oocytes than in vitrified ones (62.1 ± 3.19 and 5.48 ± 1.6%, respectively; *p* < 0.03). In general, the descriptive kinetics of development showed that the blastocysts developed from fresh oocytes occurred earlier, on average 8.5 days after the ICSI, compared with 9.3 days on average for the blastocysts developed from vitrified oocytes (Table 2).

#### 3.1.2. Effect of Sucrose, Trehalose, and Galactose as Non-Permeating CPAs during Vitrification

**Figure 2:**
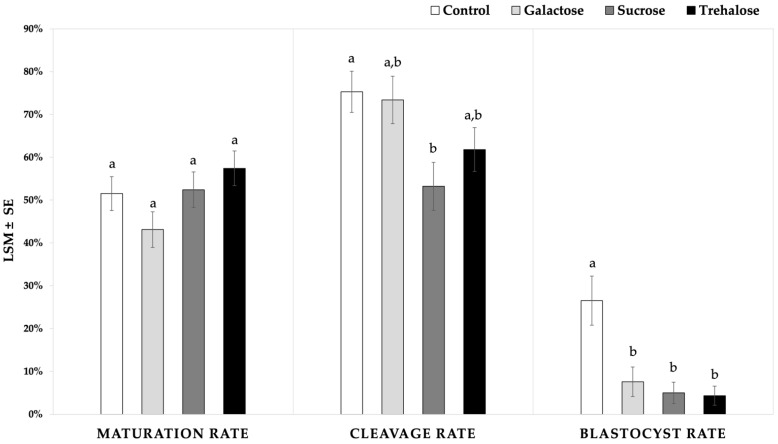
Maturation, cleavage, and blastocyst rates of immature equine oocytes vitrified with three different sugars as non-permeating CPA. Ethylene glycol and dimethyl sulfoxide were used as permeating CPAs in a 50:50 ratio; 20 and 40% (*v*/*v*) of the CPA mix were used in the equilibration and the vitrification solution, respectively. Intracytoplasmic sperm injection was performed in mature oocytes after visualization of the polar body. Cleavage and blastocyst rates represent the percentage of cleaved embryos or blastocysts, respectively, per injected oocytes. Different superscripts (a and b) represent statistical differences (*p* < 0.05) between groups. Results are expressed as least square means ± standard error (LSM ± SE).

Once the vitrification with each sugar was assessed individually, the comparison between the different sugars showed no significant difference in the maturation rate amongst the vitrification groups (sucrose: 52.4 ± 4.1%; trehalose: 57.4 ± 4.1%; galactose: 43.1 ± 4.1%), and the control (56.9 ± 4.0%; *p* > 0.05) (Figure 2). However, galactose tended to result in a lower maturation rate compared to trehalose (*p* = 0.06) and the control group (*p* = 0.069). The cleavage rates were not different either among the sugar treatments (sucrose: 53.2 ± 5.6%; trehalose: 61.8 ± 5.2%; galactose: 73.4 ± 5.5%), but the cleavage rate after the vitrification with sucrose was significantly lower than that of the control (75.3 ± 4.8%; *p* = 0.02) and tended to be lower than that of galactose (*p* = 0.06). The blastocyst rates for all the vitrified groups (sucrose: 5.0 ± 2.5%; trehalose: 4.3 ± 2.2%; galactose: 7.6 ± 3.4%) were lower compared to the control group (26.5 ± 5.7%; *p* < 0.04) (Figure 2).

### 3.2. Experiment 2: Effect of Three Different Mixtures of Permeating CPAs and Two Different Warming Regimens on Maturation, Cleavage, and Blastocyst Rates

#### 3.2.1. Comparison among All Vitrified and Non-Vitrified Oocytes

When all the vitrified oocytes, combining the results for the three CPA mixtures, are compared with fresh oocytes, the vitrified oocytes exhibited an overall reduction in the maturation, cleavage, and blastocyst rates (41.2 ± 2.1, 56.6 ± 3.0, and 6.3 ± 1.6%, respectively) when compared to fresh oocytes (58.6 ± 3.4, 71.1 ± 4.3, and 34.2 ± 5.1%, respectively; *p* < 0.01). In general, the blastocyst development from the fresh oocytes also occurred faster, on average 8 days after the ICSI, compared with 8.8 days for the blastocyst development from the oocytes vitrified with different CPAs mixtures (Table 3).

#### 3.2.2. Effect of Three Different Mixtures of Permeating CPAs on Maturation, Cleavage, and Blastocyst Rates

**Figure 3:**
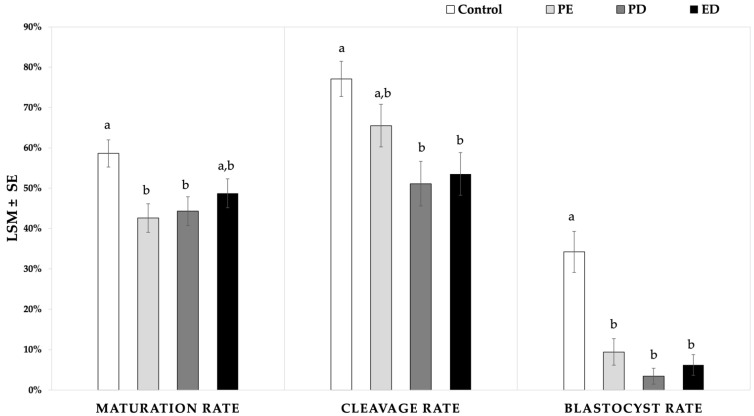
Maturation, cleavage, and blastocyst rates of immature equine oocytes vitrified with three different CPA mixtures in a 50:50 ratio. In all groups, galactose was used at 0.5 M in the vitrification solution. In the warming solution, two concentrations of galactose (0.3 vs. 0.5 mol/L) were assessed, of which the combined result was represented in this graph. Intracytoplasmic sperm injection was performed in mature oocytes after visualization of the polar body. Cleavage and blastocyst rates represent the percentage of cleaved embryos or blastocysts, respectively, per injected oocytes. Different superscripts (a and b) represent statistical differences (*p* < 0.05) between groups. Results are expressed as least square means ± standard error (LSM ± SE). ED: ethylene glycol-dimethyl sulfoxide; PE: propylene glycol-ethylene glycol; PD: propylene glycol-dimethyl sulfoxide.

In the direct comparison of the three permeating CPA combinations, the maturation rate was higher in the control (58.6 ± 3.4%) than in PD (44.3 ± 3.6%; *p* = 0.02) and PE (42.6 ± 3.5%; *p* = 0.007), while the ED reached a maturation rate comparable to the control (48.7 ± 3.6%; *p* = 0.1) (Figure 3). The cleavage rate was similar among the vitrification treatments. In comparison with the fresh oocytes, the cleavage rate in the PE (65.5 ± 5.3%) was similar to the control (77.1 ± 4.3%; *p* = 0.3), while it was significantly lower in the PD (51.1 ± 5.5%; *p* = 0.002) and ED (53.5 ± 5.3%; *p* = 0.005). The blastocyst rates were lower for all the vitrified groups (ED = 6.2 ± 2.6%, PD = 3.5 ± 2.0%, and PE = 9.4 ± 3.3%) compared to the control (34.2 ± 5.1%; *p* < 0.01) (Figure 3). The embryo development from the vitrified oocytes occurred between 8 and 9.1 days after the ICSI (PD: 8 days; PE: 8.9 days; and ED: 9.0), while the average was 8 days in the control (Table 3).

#### 3.2.3. Effect of Two Warming Regimens on Maturation, Cleavage, and Blastocyst Rates

**Figure 4:**
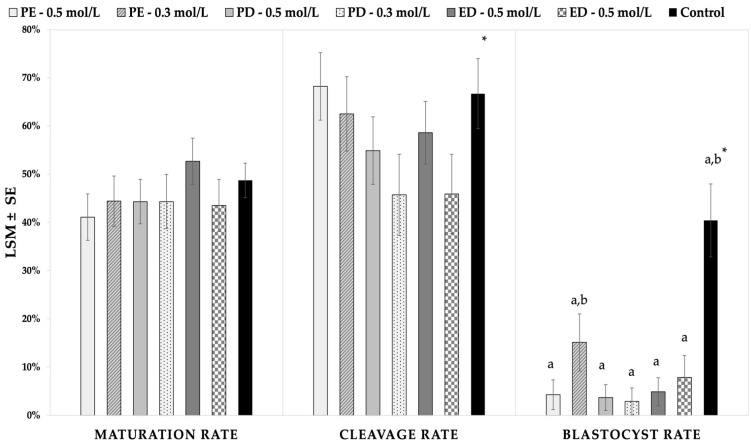
Maturation, cleavage, and blastocyst rates of immature equine oocytes vitrified with three different CPA mixtures in a 50:50 ratio (in the presence of 0.5 M galactose) and warmed in two different galactose concentrations (0.3 vs. 0.5 mol/L). Intracytoplasmic sperm injection was performed in matured oocytes after visualization of the polar body. Cleavage and blastocyst rates represent the percentage of cleaved embryos or blastocysts, respectively, per injected oocytes. Different superscripts (a and b) represent statistical›› differences (*p* < 0.05) between groups. Results are expressed as least square means ± standard error (LSM ± SE). * Values resulting from the random resampling of the control group to balance the higher number of injected oocytes compared to the vitrification treatments. PE: propylene glycol-ethylene glycol; PE: propylene glycol-dimethyl sulfoxide; PD: ethylene glycol-dimethyl sulfoxide.

First, to evaluate the effect of the warming concentration, an overall comparison between the oocytes warmed in 0.5 mol/L and 0.3 mol/L did not display differences in the maturation (46.1 ± 3.0 vs. 44.1 ± 3.1%; *p* = 0.6), cleavage (60.1 ± 4.0 vs. 51.8 ± 4.7%; *p* = 0.1), or blastocyst rates (4.6 ± 1.7% vs. 8.9 ± 2.7%; *p* = 0.2). The effect of the interaction between the mixtures of the permeating CPAs in the ES and VS and galactose concentration in the warming medium (0.3 or 0.5 mol/L) on the maturation, cleavage, and blastocyst rates are shown in Figure 4 and Supplementary Table S1. No differences in the maturation or cleavage rates were found among the vitrification groups. However, the PE-0.3 numerically had the highest blastocyst rate after vitrification (15.1%), which was not significantly lower than that of the control (*p* = 0.16), while all the other vitrification groups had significantly lower blastocyst rates compared with the control. No significant differences were obtained between the six treatment groups (Figure 4 and Table S1). The kinetics of the development of the embryos obtained from the fresh oocytes and from oocytes vitrified with different CPAs mixtures and warmed with different galactose concentrations is represented in Table 4. Although the average day of blastocyst formation seems to be faster after warming in 0.5 mol/L galactose (7.9 days) than after warming in 0.3 mol/L (9.2 days), this difference is not significant due to the low numbers of embryos (Table 4).

## 4. Discussion

This study is the first to perform a side-by-side comparison of three mixtures of permeating CPAs plus three non-permeating CPAs in equal concentrations for the vitrification of immature equine oocytes. Our study demonstrates that all the CPA mixtures used can result in blastocysts using a short vitrification protocol. Notwithstanding, the mixture of PG–EG allowed for the highest cleavage (65.5%) and blastocyst rates (9.4%) among the permeating CPAs, with a cleavage rate similar to the control (77%). In addition, the oocytes vitrified with PG–EG with 0.5 mol/L galactose and warmed in a base medium with 0.3 mol/L galactose resulted in the highest blastocyst rate (15.1%) after vitrification, representing the only group of which the blastocyst rate was not significantly lower than the control (34.2%). Moreover, galactose, a monosaccharide used with success in equine [24] and camelid [39] embryo vitrification, was tested for the first time in equine oocyte vitrification and resulted in the highest blastocyst rates among the non-permeating CPAs (7.6%), with a cleavage rate equal to the fresh oocytes.

Only a few studies have compared monosaccharides and disaccharides for oocyte or embryo vitrification, with contrasting results [34,50,51]. Kuleshova et al., 1999 [51] determined that disaccharides appear to have a greater influence on the vitrification properties of EG–saline solutions, and Huang et al., 2008 [50] showed a higher maturation rate of porcine oocytes vitrified with sucrose than those vitrified with glucose. However, McWilliam et al., 1995 [34] demonstrated a numerically greater survival rate of murine zygotes after exposure to monosaccharides than disaccharides. In studies with human [31] and pig oocytes [52], sucrose and trehalose resulted in similar maturation, cleavage, and embryo development rates. However, in our study with equine oocytes, the cleavage rate obtained with sucrose was numerically lower than that obtained with other saccharides, and was significantly lower than that of the control, while galactose gave a cleavage rate almost identical to the control. It seems that sucrose might present some degree of toxicity, as was suggested for camelid embryos [39]. In fact, in our study, both disaccharides sucrose and trehalose gave numerically lower cleavage and blastocyst rates than the monosaccharide galactose. While these differences were not statistically significant, the contrast in the cleavage rate between galactose and sucrose was very close to being significant. One of the differences of galactose versus the disaccharides is that the disaccharides displace more water than galactose due to their larger partial molar volume. The lower water ‘concentration’ (*c*_w_) results in an approximately 6% higher molal concentration of the CPAs and other solutes, and a correspondingly higher osmolality of the ES, VS, and WM when using 0.5 mol/L disaccharides compared with galactose. Further research is needed to confirm the apparent advantage of galactose and find potential mechanisms.

In humans, in which the vitrification of in vivo matured oocytes is routinely performed in clinical practice, protocols make use of a two-step increase in the CPA concentrations [53,54]. In the first step, the oocytes are incubated for a relatively long time (10–15 min) in equilibration solution, and the second step consists of exposure to the vitrification solution for 30–90 s [55,56,57]. However, for horse oocytes, despite good maturation and cleavage rates and some blastocyst development, the relatively long exposure to CPAs has been associated with CPA toxicity, affecting subsequent embryo development [11,13,58,59]. Tharasanit et al, 2006 [28] demonstrated that a short protocol did not exhibit toxicity in horse oocytes. However, this short exposure may not have provided sufficient cryoprotection since the blastocyst rate of the vitrified–warmed oocytes was low (<1%) with the lower cooling and warming rates (open-pulled straw method) that they applied. Later, studies showed that shorter CPA exposure times with minimal volume (<1 µL) could yield better blastocyst rates [12,14], and such protocols produced the only foal born to date from vitrified–warmed immature oocytes [10]. Therefore, in this study, a short vitrification protocol (less than 90 s) was selected to be optimized.

Although no significant differences among the CPAs were found in the present study, the PE mixture presented a higher cleavage rate than the other CPA combinations, with PE-0.3 also giving rise to the numerically highest blastocyst rate (15%), representing the only group not significantly different from the control. Yet, the blastocyst rate of 34.2% in the control group remains numerically higher, so further improvement of the vitrification protocol is indicated. This may be based on the use of PG–EG, which outperformed the other CPA mixtures in our study. Similarly, Canesin et al., 2017 [11] only obtained a blastocyst with the combination of PG–EG–trehalose, while this was not achieved with EG–DMSO–sucrose [11]. Furthermore, Somfai et al., 2013 [22] demonstrated in porcine oocytes that the PG–EG combination also provided greater embryo development after the vitrification of germinal vesicle-stage oocytes than the sole use of either CPA. Ethylene glycol alone or in a mixture is the most used CPA since it was demonstrated that EG is one of the safest CPAs regarding toxicity [18,60]. However, the membrane permeability of EG was found in horse oocytes to be substantially lower than that of PG [18,61]. Therefore, in short protocols, the intracellular CPA concentration will be lower when using only EG and may be insufficient to ensure cryoprotection. Conversely, PG exhibits a higher toxicity, but it presents one of the highest cell membrane permeabilities among CPAs [61]. As such, both CPAs provide complementary properties, and the combination of PG and EG can improve the cryopreservation outcome. This was supported by Somfai et al., 2015 [53], who showed that the combination of PG–EG was superior to EG–DMSO in terms of the oocyte survival after vitrification and the quality of the resulting blastocysts [52]. On the other hand, Clerico et al., 2021 [14] reported a 15% blastocyst rate, representing one of the best results with equine immature vitrified oocytes using EG–DMSO–trehalose, but these results were obtained after supplementation with the antioxidant melatonin during in vitro maturation, which improved the development after the ICSI. Using the combination of EG–DMSO—trehalose without further supplementation, they reached a blastocyst rate of 9%, which is in alignment with our blastocyst rate of 8% for the oocytes vitrified in EG-DMSO and warmed in 0.3 mol/L. Our results revealed that PG–EG provides a valuable option for equine immature oocyte vitrification since 15% was obtained when the oocytes were warmed in 0.3 mol/L, and the study of Clerico et al. [14] suggests that an even higher blastocyst yield could be obtained with the supplementation of substances that reduce stress post-vitrification/warming. However, future studies need to be performed to confirm these findings and to reveal the underlying mechanisms.

Routinely, warming is performed in a hyperosmotic solution of sugars, decreasing to an isotonic base solution in two to four steps to avoid osmotic shock or over-swelling [56,62]. However, simpler warming systems have been evaluated. Inaba et al. [63] found that warming in an isotonic solution (holding medium) was equally effective to warming in a standard hypertonic solution for in vitro bovine embryos. Later, Canesin et al., 2018 [12] warmed equine oocytes in an isotonic base solution and found similar maturation and cleavage rates compared with a base solution containing 0.4 mol/L of trehalose, but the blastocyst rate was affected in all the treatments. We evaluated the effect of lowering the galactose concentrations of the warming medium (0.3 vs. 0.5 mol/L), attempting to simplify the warming method but keeping one step to avoid osmotic shock. The galactose concentration in the WM did not affect the maturation, cleavage, and blastocyst rates, but the oocytes vitrified in the PG–EG and warmed in 0.3 mol/L resulted in the highest blastocyst rate among all the vitrification treatments in the present study (15.1%). This may indicate that a less hypertonic (or possibly even isotonic) warming medium might be beneficial for the warming of equine oocytes. The short incubation of the oocytes in the ES and in VS, and the presence of 0.5 mol/L galactose in VS limit the entry of the permeating CPAs [64], which lowers the risk of the oocytes swelling in a warming medium above their isotonic volume and reduces the need for a high sugar concentration in the WM to counter the swelling. In addition, the osmolality of the WM with 0.5 mol/L in the WM will cause the oocytes to be still strongly shrunken after equilibration in the WM, which would prolong the shrunken state of the oocytes and could possibly make the step from the WM to BM and the subsequent reswelling to isotonic volume too abrupt. The at least equal, and possibly even better, result in our study with 0.3 mol/L galactose in the WM could thus be interpreted and appear to be in line with the study of Canesin et al. [12], in which the only blastocyst resulted from the warming in the medium without sugar. More research is needed to evaluate the different warming systems for equine oocytes with gradually decreasing concentrations in order to further optimize equine oocyte vitrification.

## 5. Conclusions

In conclusion, while we were able to produce blastocysts after the vitrification of equine immature oocytes with all six CPAs, the overall developmental competence remained lower compared to the fresh control. The PE mixture presented the highest cleavage rate compared with the other CPA combinations, while the PE-0.3 also gave rise to a competitive blastocyst rate of 15%, representing the only treatment not significantly lower than the fresh control. Moreover, galactose, a monosaccharide tested for the first time in equine oocyte vitrification, resulted in the highest blastocyst rates after vitrification as well as in cleavage rates equal to those of the control. As such, PE–galactose provides a valuable option for equine immature oocyte vitrification and should be considered as an alternative for the future optimization of the vitrification protocols for equine immature oocytes.

## Figures and Tables

**Table 1:** Composition of solutions and exposure times to cryoprotectants.

Experiment	Group	ES	VS	WS
Experiment 1.	Sucrose	BS + ED 20%	BS + ED 40% + **Sucrose 0.5 M**	BS + **Sucrose 0.5 M**
	Trehalose		BS + ED 40% + **Trehalose 0.5 M**	BS + **Trehalose 0.5 M**
	Galactose		BS + ED 40% + **Galactose 0.5 M**	BS + **Galactose 0.5 M**
Experiment 2.	EG − DMSO (ED-0.5)	BS + **ED** 20%	BS + **ED 40%** + Galactose 0.5 M	BS + **Galactose 0.5 M**
	EG − DMSO (ED-0.3)			BS + **Galactose 0.3 M**
	PG − EG (PE-0.5)	BS + **PE** 20%	BS + **PE 40%** + Galactose 0.5 M	BS + **Galactose 0.5 M**
	PG − EG (PE-0.3)			BS + **Galactose 0.3 M**
	PG − DMSO (PD-0.5)	BS + **PD** 20%	BS + **PD 40%** + Galactose 0.5 M	BS + **Galactose 0.5 M**
	PG − DMSO (PD-0.3)			BS + **Galactose 0.3 M**

The same base solution (BS) was used for all treatments: Tissue Culture Medium-199 (TCM-199) with Hanks’ salts with 0.4% (*w*/*v*) bovine serum albumin (BSA) (A6003). For vitrification, oocytes were held 25 s in equilibration solution (ES), immersed in vitrification solution (VS) for 15 s, loaded onto the cryo-device, and plunged in liquid nitrogen; for warming, oocytes were immersed in warming solution (WS) for 5 min, held in BS until warming of a complete group was finished, and then immediately moved to in vitro maturation medium. EG: ethylene glycol; DMSO: dimethyl sulfoxide; PG: propylene glycol; ED: ethylene glycol-dimethyl sulfoxide; PE: propylene glycol-ethylene glycol; PD: propylene glycol-dimethyl sulfoxide; M: molar (mol/L). Percentage of permeating CPA mixtures in a 50:50 ratio. Font in bold within the description of the solutions indicate where there are differences between treatments.

**Table 2:** Descriptive kinetics of development of embryos obtained from fresh oocytes and oocytes vitrified with different sugars.

Group	Injected Oocytes	Day 7	Day 8	Day 9	Day 10	Total	Average Day
Control	80	5	5	7	4	21	8.5
Galactose	64		1	1	3	5	9.4
Sucrose	79			2	2	4	9.5
Trehalose	89		1	2	1	4	9.0

Results express the number of blastocysts obtained each day per group (control and each sugar), with Day 0 representing the day of ICSI.

**Table 3:** Descriptive kinetics of development of embryos obtained from fresh and vitrified oocytes with different CPAs mixtures.

Group	Injected Oocytes	Day 7	Day 8	Day 9	Day 10	Total	Average Day
Control	117	15	5	10	3	33	8.0
PE	85	2	1	1	4	8	8.9
PD	73	1	0	1	0	2	8.0
ED	95	1	2	0	4	7	9.0

Results express the number of blastocysts obtained each day per group (control and each CPA mixture). ED: ethylene glycol-dimethyl sulfoxide; PE: propylene glycol-ethylene glycol; PD: propylene glycol-dimethyl sulfoxide.

**Table 4:** Descriptive kinetics of development of embryos obtained from fresh oocytes and from oocytes vitrified with different CPAs mixtures and warmed with different galactose concentrations.

CPA Mixture	Warming Concentration	Injected Oocytes	Day 7	Day 8	Day 9	Day 10	Total	Average Day
PE	0.5 mol/L	40	2				2	7.0
	0.3 mol/L	44		1	1	4	6	9.5
PD	0.5 mol/L	51	1		1		2	8.0
	0.3 mol/L	35			1		1	9.0
ED	0.5 mol/L	58	1	1		1	3	8.3
	0.3 mol/L	38		1		2	3	9.5
Control		110	15	5	10	3	33	8.0

Results express the number of embryos obtained each day per group (control and each mixture warmed in different galactose concentration). PE: propylene glycol-ethylene glycol: PE; propylene glycol-dimethyl sulfoxide: PD; ethylene glycol-dimethyl sulfoxide.

## Data Availability

Not applicable.

## Supplementary Materials

The following are available online at https://www.mdpi.com/article/10.3390/ani11113077/s1. Table S1. Rates of in vitro maturation, cleavage, and blastocyst formation after ICSI of vitrified–warmed immature equine COCs according to the CPA mixture and the galactose concentration in the warming medium.

## References

[1] Claes A., Cuervo-Arango J., van den Broek J., Galli C., Colleoni S., Lazzari G., Deelen C., Beitsma M., Stout T.A. (2019). Factors affecting the likelihood of pregnancy and embryonic loss after transfer of cryopreserved in vitro produced equine embryos. Equine Vet. J..

[2] Lazzari G., Colleoni S., Crotti G., Turini P., Fiorini G., Barandalla M., Landriscina L., Dolci G., Benedetti M., Duchi R. (2020). Laboratory Production of Equine Embryos. J. Equine Vet. Sci..

[3] Gambini A., Maserati M. (2018). A journey through horse cloning. Reprod. Fertil. Dev..

[4] Papas M., Govaere J., Peere S., Gerits I., Van de Velde M., Angel-Velez D., De Coster T., Van Soom A., Smits K. (2021). Anti-Müllerian Hormone and OPU-ICSI Outcome in the Mare. Animals.

[5] De Coster T., Angel-Velez D., Van Soom A., Woelders H., Smits K. (2020). Cryopreservation of equine oocytes: Looking into the crystal ball. Reprod. Fertil. Dev..

[6] Hinrichs K. (2018). Assisted reproductive techniques in mares. Reprod. Domest. Anim..

[7] Smits K., Hoogewijs M., Woelders H., Daels P., Van Soom A. (2012). Breeding or Assisted Reproduction? Relevance of the Horse Model Applied to the Conservation of Endangered Equids. Reprod. Domest. Anim..

[8] Arav A. (2014). Cryopreservation of oocytes and embryos. Theriogenology.

[9] Maclellan L.J., Carnevale E.M., Silva M.A.C., Scoggin C.F., Bruemmer J.E., Squires E.L. (2002). Pregnancies from vitrifed equine oocytes collected from super-stimulated and non-stimulated mares. Theriogenology.

[10] Ortiz-Escribano N., Bogado Pascottini O., Woelders H., Vandenberghe L., De Schauwer C., Govaere J., Van den Abbeel E., Vullers T., Ververs C., Roels K. (2018). An improved vitrification protocol for equine immature oocytes, resulting in a first live foal. Equine Vet. J..

[11] Canesin H.S., Brom-de-Luna J.G., Choi Y.H., Ortiz I., Diaw M., Hinrichs K. (2017). Blastocyst development after intracytoplasmic sperm injection of equine oocytes vitrified at the germinal-vesicle stage. Cryobiology.

[12] Canesin H.S., Brom-de-Luna J.G., Choi Y.-H., Pereira A.M., Macedo G.G., Hinrichs K. (2018). Vitrification of germinal-vesicle stage equine oocytes: Effect of cryoprotectant exposure time on in-vitro embryo production. Cryobiology.

[13] Angel D., Canesin H.S., Brom-de-Luna J.G., Morado S., Dalvit G., Gomez D., Posada N., Pascottini O.B., Urrego R., Hinrichs K. (2020). Embryo development after vitrification of immature and in vitro-matured equine oocytes. Cryobiology.

[14] Clérico G., Taminelli G., Veronesi J.C., Polola J., Pagura N., Pinto C., Sansinena M. (2021). Mitochondrial function, blastocyst development and live foals born after ICSI of immature vitrified/warmed equine oocytes matured with or without melatonin. Theriogenology.

[15] Paredes E., Mazur P. (2013). The survival of mouse oocytes shows little or no correlation with the vitrification or freezing of the external medium, but the ability of the medium to vitrify is affected by its solute concentration and by the cooling rate. Cryobiology.

[16] Fahy G.M., Levy D.I., Ali S.E. (1987). Some emerging principles underlying the physical properties, biological actions, and utility of vitrification solutions. Cryobiology.

[17] Shaw J.M., Jones G.M. (2003). Terminology associated with vitrification and other cryopreservation procedures for oocytes and embryos. Hum. Reprod. Update.

[18] Best B.P. (2015). Cryoprotectant Toxicity: Facts, Issues, and Questions. Rejuvenation Res..

[19] Rall W.F., Fahy G.M. (1985). Ice-free cryopreservation of mouse embryos at −196 degrees C by vitrification. Nature.

[20] Rall W.F. (1987). Factors affecting the survival of mouse embryos cryopreserved by vitrification. Cryobiology.

[21] Szurek E.A., Eroglu A. (2011). Comparison and Avoidance of Toxicity of Penetrating Cryoprotectants. PLoS ONE.

[22] Somfai T., Nakai M., Tanihara F., Noguchi J., Kaneko H., Kashiwazaki N., Egerszegi I., Nagai T., Kikuchi K. (2013). Comparison of ethylene glycol and propylene glycol for the vitrification of immature porcine oocytes. J. Reprod. Dev..

[23] Hurtt A., Ladim-Alverenga F., Seidel J., Squires E. (2000). Vitrification of immature and mature equine and bovine oocytes in an ethylene glycol, ficoll and sucrose solution using open-pulled straws. Theriogenology.

[24] Hunt C.J. (2017). Cryopreservation: Vitrification and Controlled Rate Cooling. Methods Mol. Biol..

[25] Kasai M. (1997). Vitrification: Refined Strategy for the Cryopreservation of Mammalian Embryos. J. Mamm. Ova Res..

[26] Yavin S., Arav A. (2007). Measurement of essential physical properties of vitrification solutions. Theriogenology.

[27] Tharasanit T., Colenbrander B., Stout T.A.E. (2006). Effect of maturation stage at cryopreservation on post-thaw cytoskeleton quality and fertilizability of equine oocytes. Mol. Reprod. Dev..

[28] Tharasanit T., Colleoni S., Lazzari G., Colenbrander B., Galli C., Stout T.A.E. (2006). Effect of cumulus morphology and maturation stage on the cryopreservability of equine oocytes. Reproduction.

[29] Tharasanit T., Colleoni S., Galli C., Colenbrander B., Stout T.A.E. (2009). Protective effects of the cumulus-corona radiata complex during vitrification of horse oocytes. Reproduction.

[30] Maclellan L.J., Lane M., Sims M., Squires E.L. (2001). Effect of sucrose or threalos on vitrification of equine oocytes 12 h or 24 h after the onset of maturation. Theriogenology.

[31] Coello A., Campos P., Remohí J., Meseguer M., Cobo A. (2016). A combination of hydroxypropyl cellulose and trehalose as supplementation for vitrification of human oocytes: A retrospective cohort study. J. Assist. Reprod. Genet..

[32] Zhang Z., Wang T., Hao Y., Panhwar F., Chen Z., Zou W., Ji D., Chen B., Zhou P., Zhao G. (2017). Effects of trehalose vitrification and artificial oocyte activation on the development competence of human immature oocytes. Cryobiology.

[33] Lestari S.W., Ilato K.F., Pratama M.I.A., Fitriyah N.N., Pangestu M., Pratama G., Margiana R. (2018). Sucrose ‘Versus’ Trehalose Cryoprotectant Modification in Oocyte Vitrification: A Study of Embryo Development. Biomed. Pharmacol. J..

[34] McWilliams R.B., Gibbons W.E., Leibo S.P. (1995). Fertilization and early embryology: Osmotic and physiological responses of mouse zygotes and human oocytes to mono- and disaccharides. Hum. Reprod..

[35] Checura C.M., Seidel G.E. (2007). Effect of macromolecules in solutions for vitrification of mature bovine oocytes. Theriogenology.

[36] Herrick J.R., Wang C., Machaty Z. (2016). The effects of permeating cryoprotectants on intracellular free-calcium concentrations and developmental potential of in vitro-matured feline oocytes. Reprod. Fertil. Dev..

[37] Campos-Chillòn L.F., Suh T.K., Barcelo-Fimbres M., Seidel G.E., Carnevale E.M. (2009). Vitrification of early-stage bovine and equine embryos. Theriogenology.

[38] Kobayashi S., Takei M., Kano M., Tomita M., Leibo S.P. (1998). Piglets Produced by Transfer of Vitrified Porcine Embryos after Stepwise Dilution of Cryoprotectants. Cryobiology.

[39] Herrid M., Billah M., Skidmore J.A. (2017). Successful pregnancies from vitrified embryos in the dromedary camel: Avoidance of a possible toxic effect of sucrose on embryos. Anim. Reprod. Sci..

[40] Lutz J.C., Johnson S.L., Duprey K.J., Taylor P.J., Vivanco-Mackie H.W., Ponce-Salazar D., Miguel-Gonzales M., Youngs C.R. (2020). Birth of a Live Cria After Transfer of a Vitrified-Warmed Alpaca (Vicugna pacos) Preimplantation Embryo. Front. Vet. Sci..

[41] Choi Y.H., Velez I.C., Riera F.L., Roldán J.E., Hartman D.L., Bliss S.B. (2011). Successful cryopreservation of expanded equine blastocysts. Theriogenology.

[42] Choi Y.-H., Hinrichs K. (2017). Vitrification of in vitro-produced and in vivo-recovered equine blastocysts in a clinical program. Theriogenology.

[43] Griveau J.F., Lopes M., Jouve G., Veau S., Ravel C., Morcel K. (2015). Vitrification: Principles and results. J. Gynecol. Obstet. Biol. Reprod. Paris.

[44] Vajta V.G., Nagy Z.P. (2006). Are programmable freezers still needed in the embryo laboratory? Review on vitrification. Reprod. Biomed. Online.

[45] Li W., Zhou X., Wang H., Liu B. (2012). Numerical analysis to determine the performance of different oocyte vitrification devices for cryopreservation. Cryo-Letters.

[46] Kasai M., Zhu S.E., Pedro P.B., Nakamura K., Sakurai T., Edashige K. (1996). Fracture damage of embryos and its prevention during vitrification and warming. Cryobiology.

[47] Chian R. (2014). Oocyte vitrification: Advances, progress and future goals. J. Assist. Reprod. Genet..

[48] Fuller B., Paynter S. (2004). Fundamentals of cryobiology in reproductive medicine. Reprod. Biomed. Online.

[49] Jackowski S., Leibo S.P., Mazur P. (1980). Glycerol permeabilities of fertilized and infertilized mouse ova. J. Exp. Zool..

[50] Huang J., Li Q., Zhao R., Li W., Han Z., Chen X., Xiao B., Wu S., Jiang Z., Hu J. (2008). Effect of sugars on maturation rate of vitrified-thawed immature porcine oocytes. Anim. Reprod. Sci..

[51] Kuleshova L.L., Macfarlane D.R., Trounson A.O., Shaw J.M. (1999). Sugars exert a major influence on the vitrification properties of ethylene glycol-based solutions and have low toxicity to embryos and oocytes. Cryobiology.

[52] Somfai T., Men N.T., Noguchi J., Kaneko H., Kashiwazaki N., Kikuchi K. (2015). Optimization of cryoprotectant treatment for the vitrification of immature cumulus-enclosedporcine oocytes: Comparison of sugars, combinations of permeating cryoprotectants and equilibrationregimens. J. Reprod. Dev..

[53] Youm H.S., Choi J.-R., Oh D., Rho Y.H. (2018). Survival Rates in Closed and Open Vitrification for Human Mature Oocyte Cryopreservation: A Meta-Analysis. Gynecol. Obstet. Invest..

[54] Kuwayama M. (2007). Highly efficient vitrification for cryopreservation of human oocytes and embryos: The Cryotop method. Theriogenology.

[55] Mandawala A.A., Harvey S.C., Roy T.K., Fowler K.E. (2016). Cryopreservation of animal oocytes and embryos: Current progress and future prospects. Theriogenology.

[56] Kuwayama M., Vajta G., Kato O., Leibo S.P. (2005). Highly efficient vitrification method for cryopreservation of human oocytes. Reprod. Biomed. Online.

[57] Rienzi L., Gracia C., Maggiulli R., LaBarbera A.R., Kaser D.J., Ubaldi F.M., Vanderpoel S., Racowsky C. (2017). Oocyte, embryo and blastocyst cryopreservation in ART: Systematic review and meta-analysis comparing slow-freezing versus vitrification to produce evidence for the development of global guidance. Hum. Reprod. Update.

[58] Carboni S., Rosati I., Lj M., Ariu F., Bogliolo L., Mt Z., Pau S., Em C., Ledda S. Vitrification of gv and ivm horse oocytes with two different equilibration methods. Proceedings of the 10th congress of Italian Society of Animal Reproduction (SIRA).

[59] De Leon P.M.M., Campos V.F., Corcini C.D., Santos E.C.S., Rambo G., Lucia T., Deschamps J.C., Collares T. (2012). Cryopreservation of immature equine oocytes, comparing a solid surface vitrification process with open pulled straws and the use of a synthetic ice blocker. Theriogenology.

[60] Cobo A., Diaz C. (2011). Clinical application of oocyte vitrification: A systematic review and meta-analysis of randomized controlled trials. Fertil. Steril..

[61] Lotz J., Içli S., Liu D., Caliskan S., Sieme H., Wolkers W.F., Oldenhof H. (2021). Transport processes in equine oocytes and ovarian tissue during loading with cryoprotective solutions. Biochim. Biophys. Acta-Gen. Subj..

[62] Parmegiani L., Tatone C., Cognigni G.E., Bernardi S., Troilo E., Arnone A., Maccarini A.M., Di Emidio G., Vitti M., Filicori M. (2014). Rapid warming increases survival of slow-frozen sibling oocytes: A step towards a single warming procedure irrespective of the freezing protocol?. Reprod. Biomed. Online.

[63] Inaba Y., Aikawa Y., Hirai T., Hashiyada Y., Yamanouchi T., Misumi K., Ohtake M., Somfai T., Kobayashi S., Saito N. (2011). In-straw cryoprotectant dilution for bovine embryos vitrified using Cryotop. J. Reprod. Dev..

[64] Woelders H., Guignot F., Ortiz-Escribano N., van Soom A., Smits K. (2018). Simulations of osmotic events in vitrification of equine oocytes and porcine embryos. Cryobiology.

