# Supplementary material for: New Alternative Mixtures of Cryoprotectants for Equine Immature Oocyte Vitrification
Source: Animals (Basel). 2021 Oct 28;11(11):3077. doi: 10.3390/ani11113077 (PMC8614364; doi:10.3390/ani11113077)
Supplement: Supplementary file 1 [file animals-11-03077-s001.zip › animals-1422625-supplementary.pdf]

Table S1. Rates of in vitro maturation, cleavage, and blastocyst formation after ICSI of vitrified–warmed immature equine COCs according to the CPA mixture and the galactose concentration in the warming medium.

**CPA Mixture**	**Warming Concentration**	*n*	**Mature (LSM ± SE)**	**Cleaved (LSM ± SE)**	**Blastocyst (LSM ± SE)**
PE	0.5 mol/L	107	40 (41.1 ± 4.8%)	30 (68.2 ± 7.0%)	2 (4.3 ± 3.1%) [a]
	0.3 mol/L	90	44 (44.4% ± 5.2%)	25 (62.5 ± 7.7%)	6 (15.1 ± 5.9%) [a,b]
PD	0.5 mol/L	115	51 (44.3% ± 4.6%)	28 (54.9 ± 7.0%)	2 (3.7 ± 2.7%) [a]
	0.3 mol/L	79	35 (44.3% ± 5.6%)	16 (45.7 ± 8.4%)	1 (2.9 ± 2.8%) [a]
ED	0.5 mol/L	110	58 (52.7% ± 4.8%)	34 (58.6 ± 6.5%)	3 (4.9 ± 2.9%) [a]
	0.3 mol/L	85	38 (43.5% ± 5.4%)	17 (45.9 ± 8.2%)	3 (7.9 ± 4.5%) [a]
Control		183	110 (48.7 ± 3.6%)	65 (66.7 ± 7.27%) *	33 (40.4 ± 7.57) [b*]

Maturation, cleavage, and blastocyst rates of immature equine oocytes vitrified with three different CPA mixtures in a 50:50 ratio (in the presence of 0.5 M galactose) and warmed in two different galactose concentrations (0.3 vs. 0.5 mol/L). Intracytoplasmic sperm injection was performed in matured oocytes after visualization of the polar body. Cleavage and blastocyst rates represent the percentage of cleaved embryos or blastocysts, respectively, per injected oocytes. Different superscripts (a and b) represent statistical differences ($p < 0.05$) between groups. Results are expressed as least square means ± standard error (LSM ± SE). * a,b values resulting from the random resampling of the control group to balance the higher number of injected oocytes compared to the vitrification treatments. PE: propylene glycol–ethylene glycol: PE; propylene glycol–dimethyl sulfoxide: PD; ethylene glycol–dimethyl sulfoxide: ED.
